# Supplementary figures and images for: Contractile and Mechanical Properties of Epithelia with Perturbed Actomyosin Dynamics
Source: PLoS One. 2014 Apr 23;9(4):e95695. doi: 10.1371/journal.pone.0095695 (PMC3997421; doi:10.1371/journal.pone.0095695)

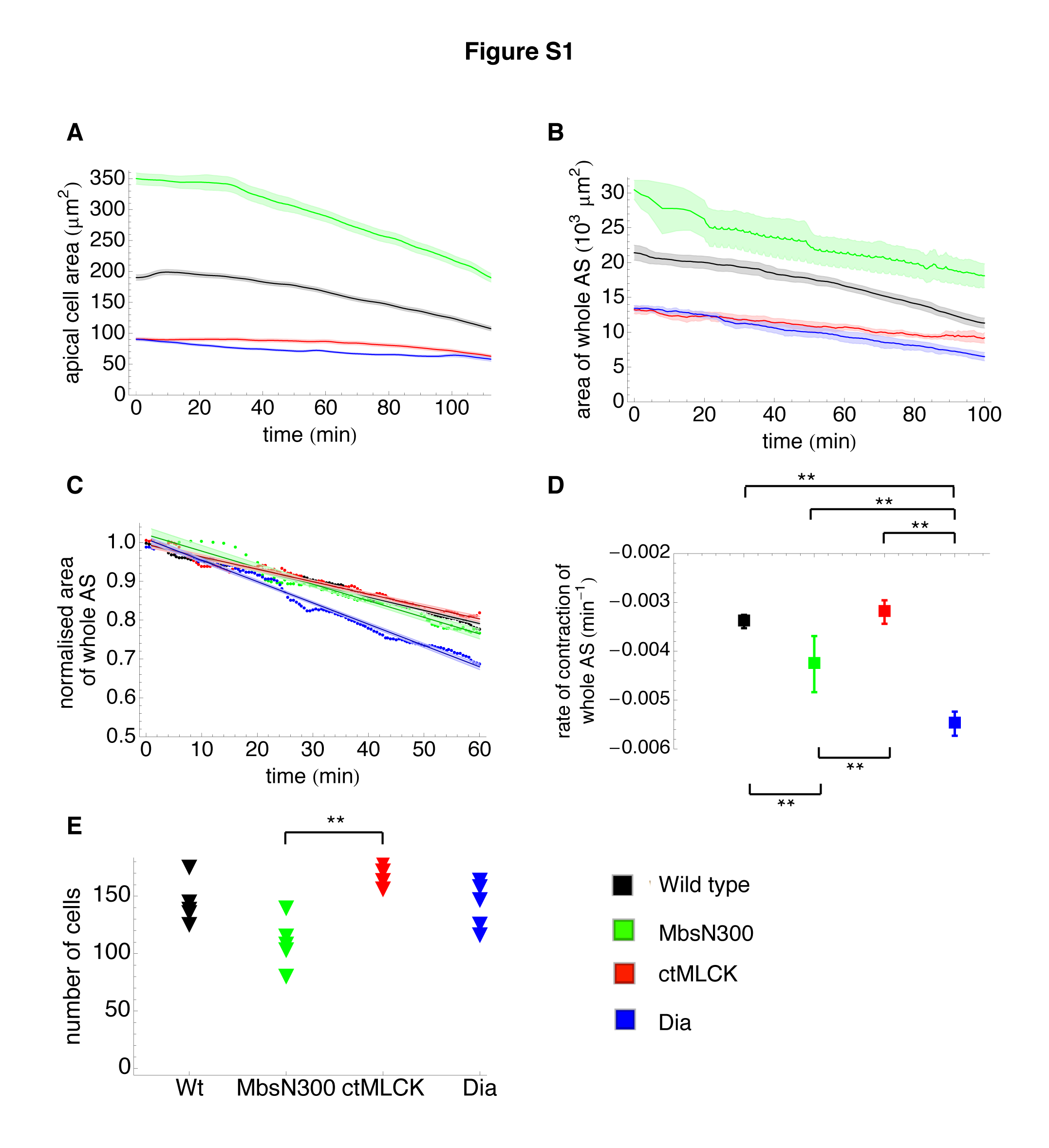

Supplement: Figure S1 — Cell and tissue behaviour of the whole AS. (A) Apical cell area over time for wild type –ASGal4, ECadGFP- (black, pooled from five embryos), ASGal4/UASMbsN300 (green, pooled from five embryos), ASGal4/UASctMLCK (red, pooled from four embryos), and ASGal4/UAS-DiaCA embryos (blue, pooled from five embryos). Shaded regions represent the standard error of the mean. (B) Mean absolute area of the AS over 100 min. Shaded regions represent the standard error of the mean. (data for wild type was pooled from five embryos, for ASGal4/UASMbsN300 from five embryos, for ASGal4/UASctMLCK from four embryos, for ASGal4/UASDiaCA from three embryos) (C) Overlay of dotted lines representing the mean area normalised to the mean area at time 0 min for 60 min (slow phase) and fitted lines with mean prediction bands of confidence level 0.95. (D) Slopes of the fitted lines in B presented as mean with 99% confidence intervals adjusted with the Bonferroni method. Significant differences were determined by comparison of the confidence intervals and are indicated by stars (**p<0.01). (E) Number of cells in the AS. Each triangle represents one embryo. Significant differences were determined by a Student's t-test with a Holm's correction and are indicated by stars (**p<0.01). (TIF) [file pone.0095695.s001.tif]

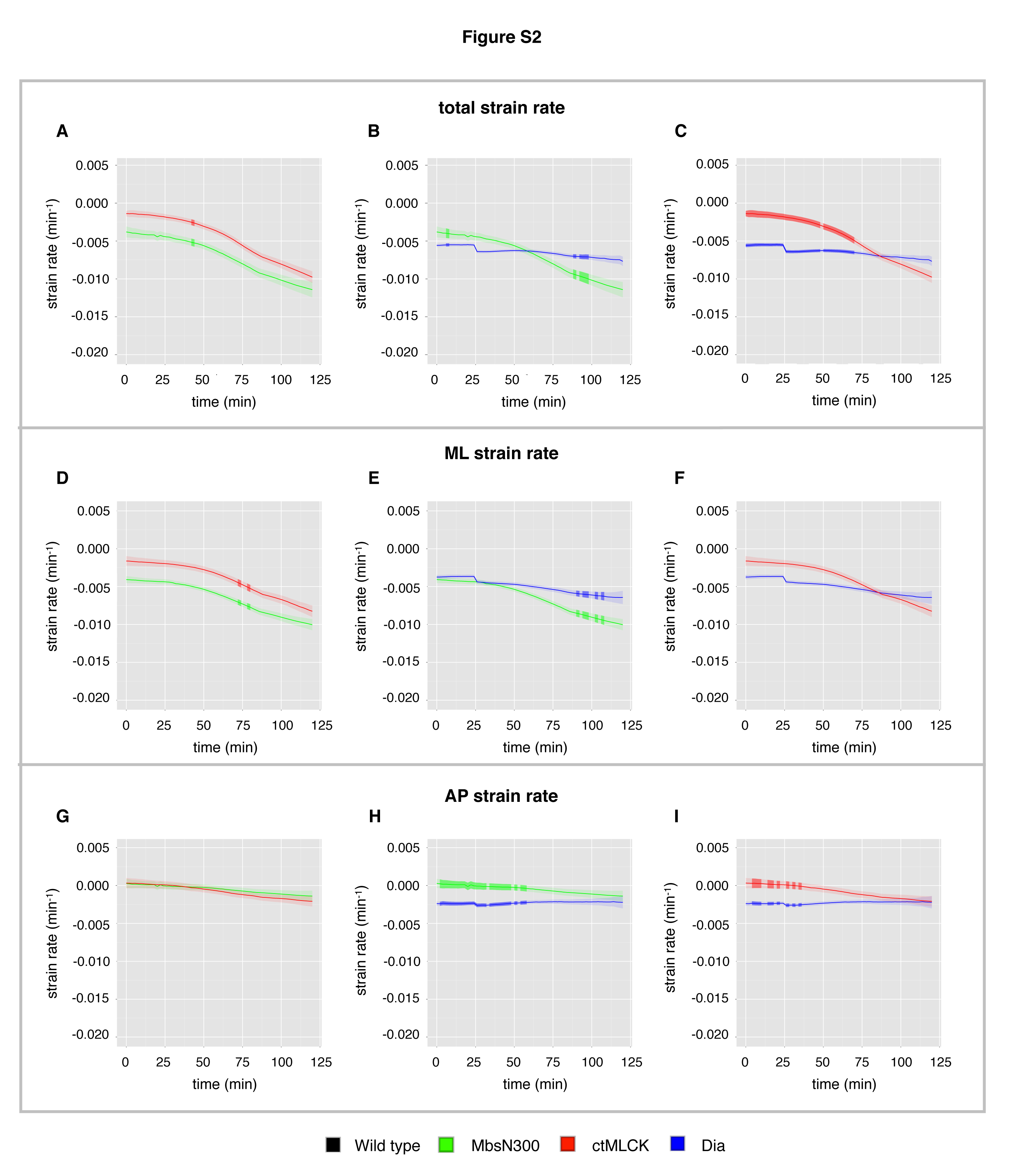

Supplement: Figure S2 — Pairwise comparisons of average cell strain rates between the different genotypes. Pairwise comparisons of total strain rate (A–C), ML strain rate (D–F), and AP strain rate (G–I) between ASGal4/UASMbsN300 and ASGal4/UASctMLCK embryos (A, D, G), ASGal4/UASMbsN300 and ASGal4/UASDiaCA embryos (B, E, H) and between ASGal4/UASctMLCK, and ASGal4/UASDiaCA embryos (C, F. I) (data for wild type was pooled from five embryos, for ASGal4/UASMbsN300 from five embryos, for ASGal4/UASctMLCK from four embryos, for ASGal4/UASDiaCA from five embryos). Shaded ribbons straddling average strain rates represent a combined mean within-experiment variance plus the variance of experimental means, while darker shaded ribbons represent epochs where one genotype behaviour was significantly different from the other according to linear mixed effects models. (TIF) [file pone.0095695.s002.tif]

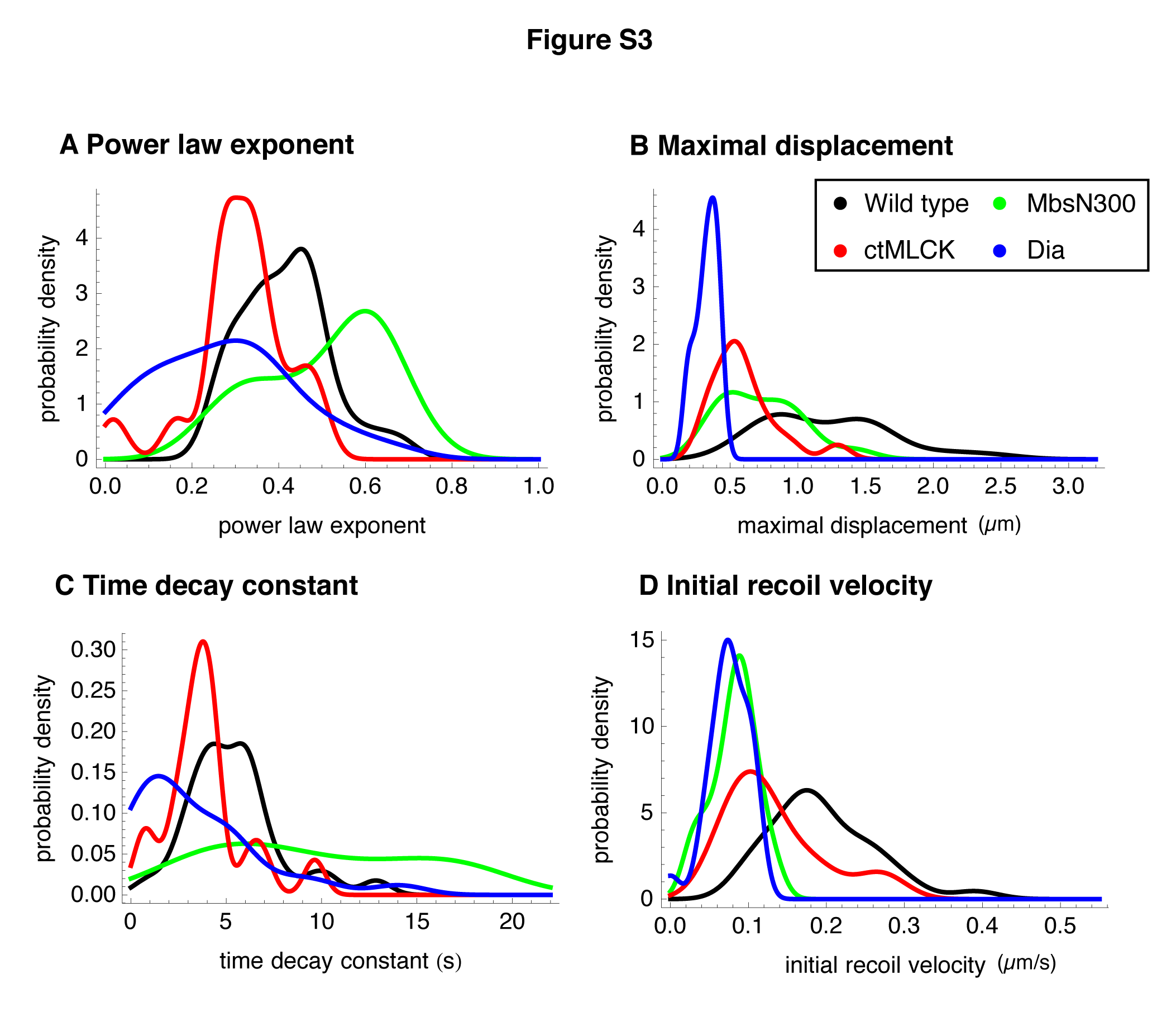

Supplement: Figure S3 — Parameters for local response to laser ablation. We show the kernel density estimates for the power law exponent (A), the maximal displacement (B), the time decay constant (C), and the initial recoil velocity (D). (TIF) [file pone.0095695.s003.tif]

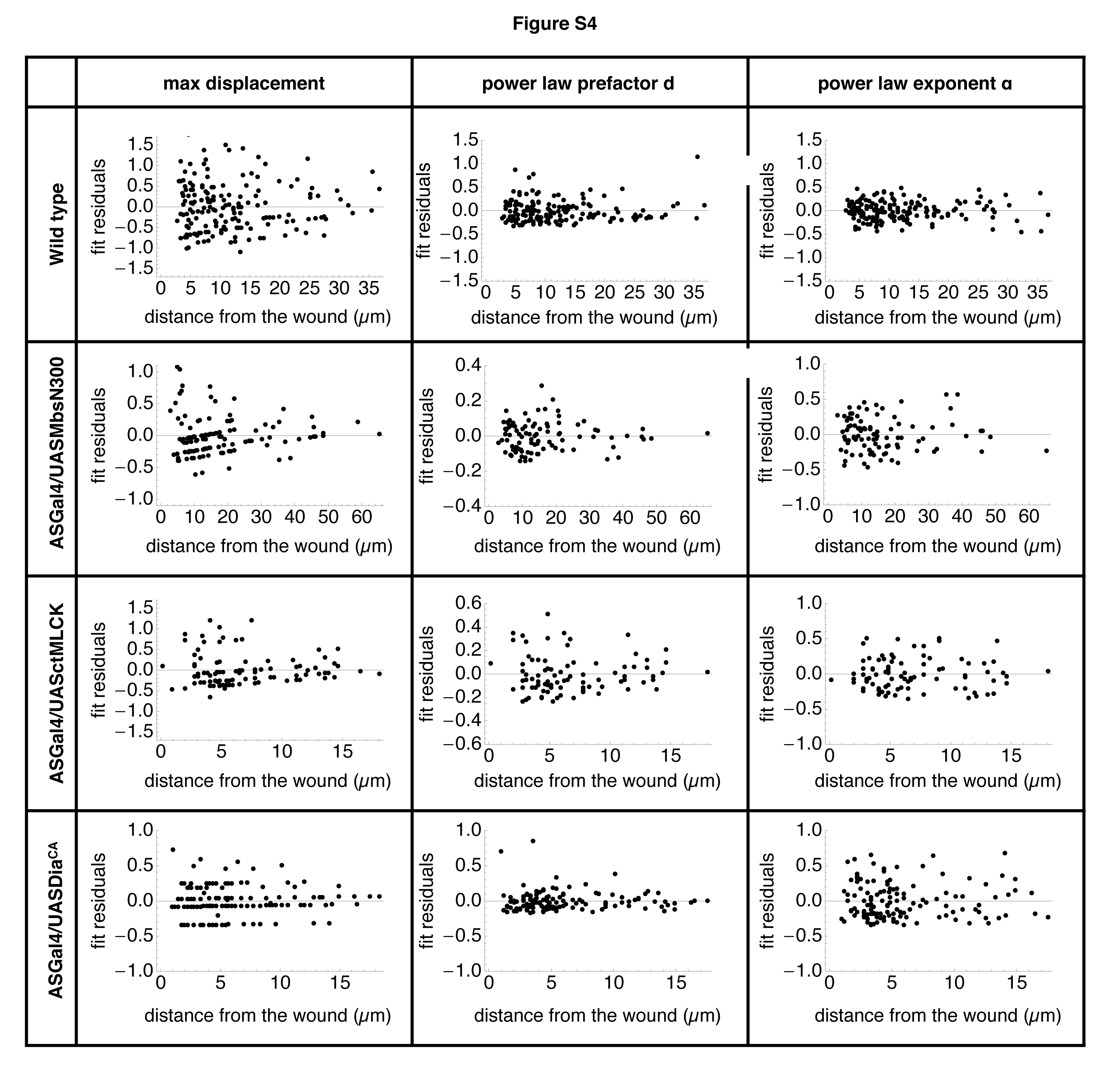

Supplement: Figure S4 — Residuals for the fitting of the tissue scale behaviour of wild type embryos and embryos with perturbed actin or myosin dynamics. The random distributions of the residuals (difference between the fitted curve and the data) indicate that the linear curve fits the different parameters and genotypes equally well. (TIF) [file pone.0095695.s004.tif]

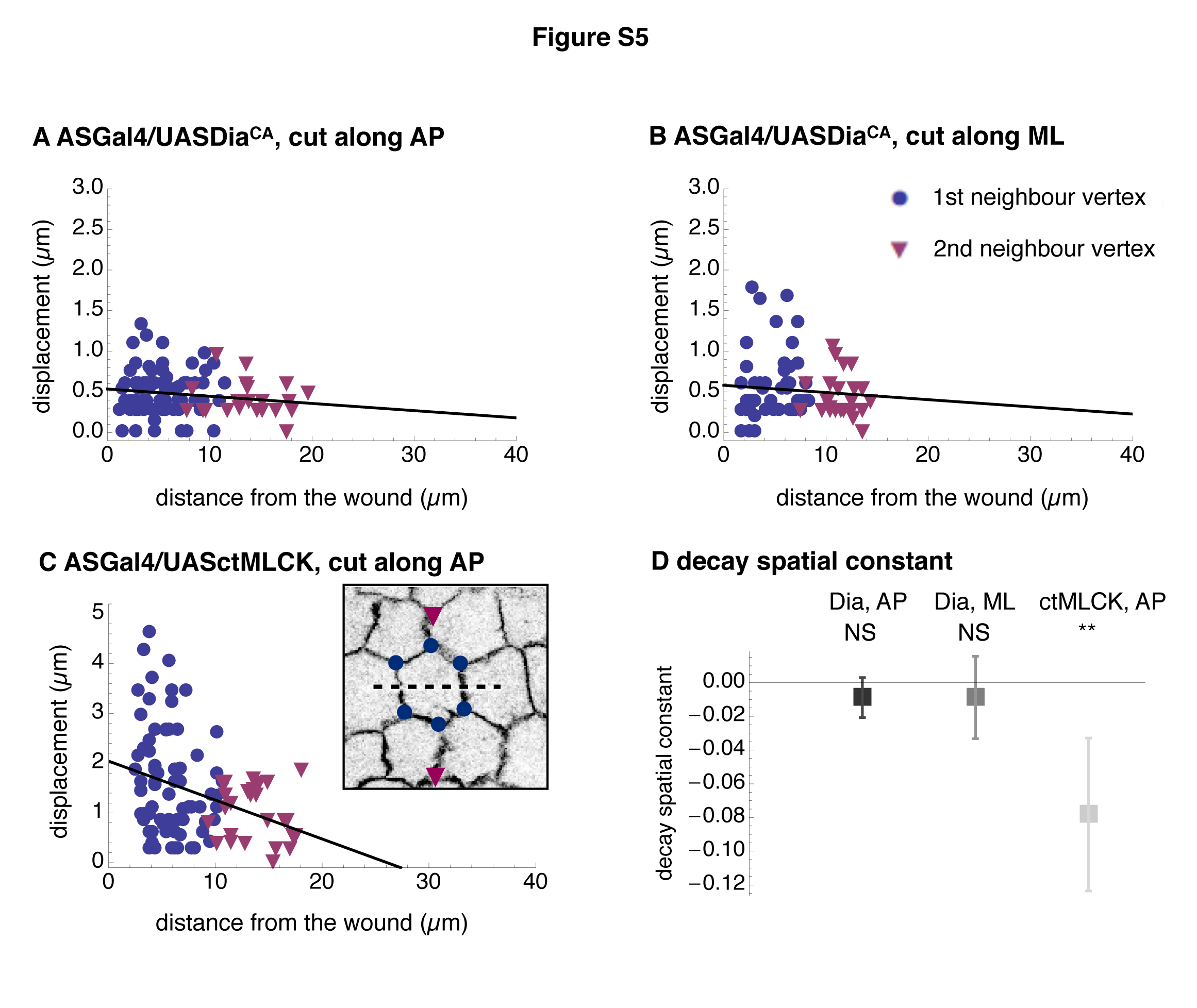

Supplement: Figure S5 — Tissue scale behaviour. Maximal displacement after laser ablation performed with increased laser power of cell vertices surrounding the wound as a function of their distance to the wound for cuts parallel to the anterior-posterior axis in ASGal4/UASDiaCA embryos (17 embryos/99 vertices) (A), cuts parallel to the dorsal-ventral axis in ASGal4/UASDiaCA (12 embryos/71 vertices) (B), and cuts parallel to the anterior-posterior axis in ASGal4/UASctMLCK (21 embryos/100 vertices) (C). Note the difference in y-axis range between A, B and C. The maximal displacement of the vertices surrounding the laser cut and 2nd neighbours are shown as disks and triangles, respectively. (D) The decay spatial constant is shown as mean with error bars indicating a 95% confidence interval. Stars indicate significant difference from 0 (**p<0.01). (TIF) [file pone.0095695.s005.tif]
